# High-Intensity Interval Training, but Not Whole-Body Cryostimulation, Affects Bone-Mechanosensing Markers and Induces the Expression of Differentiation Markers in Osteoblasts Cultured with Sera from Overweight-to-Obese Subjects

**DOI:** 10.3390/jpm14101015

**Published:** 2024-09-24

**Authors:** Marta Gomarasca, Ewa Ziemann, Veronica Sansoni, Marta Flis, Silvia Perego, Joanna Jaworska, Laura Gerosa, Martina Faraldi, Giovanni Lombardi

**Affiliations:** 1 Laboratory of Experimental Biochemistry and Molecular Biology, IRCCS Ospedale Galeazzi-Sant’Ambrogio, 20157 Milano, Italy; marta.gomarasca@grupposandonato.it (M.G.); silvia.perego@hotmail.it (S.P.); laura.gerosa@grupposandonato.it (L.G.); martina.faraldi@grupposandonato.it (M.F.); giovanni.lombardi@grupposandonato.it (G.L.); 2 Department of Athletics, Strength and Conditioning, Poznan University of Physical Education, 61-871 Poznan, Poland; ziemann@awf.poznan.pl; 3 Department of Physiology, Gdansk University of Physical Education and Sport, 80-854 Gdansk, Poland; marta.kozlowska@awf.gda.pl; 4 Department of Physiology, Medical University of Gdansk, 80-854 Gdansk, Poland; joanna.jaworska@gumed.edu.pl

**Keywords:** cryostimulation, physical activity, resistance training, bone markers, osteogenic differentiation, obesity

## Abstract

**Background/Objectives:** Although there have been some clinical observations made, the mechanistic effects on bone metabolism of whole-body cryostimulation and high-intensity interval training (HIIT), either alone or in combination, are still debated. Here, we have investigated their effects on circulating osteo-immune and bone metabolic markers (osteopontin, osteocalcin, sclerostin, dikkopf-related protein 1, and fibroblast-growth factor 23) and their potential effects on osteoblast differentiation and function, *in vitro*, by treating SaOS-2 osteoblast-like cells with the sera obtained from the subjects who had undergone the different interventions or untreated control subjects. **Methods:** Sixty-seven inactive, overweight-to-obese participants (body mass index = 31.9 ± 5.0 kg·m^−2^, 42 ± 13 years old) were recruited and randomly assigned to one group: control (CTRL, n = 14), training (HIIT, 6 sessions, n = 13), WBC (CRYO, 10 sessions, n = 17) or training combined with WBC (CRYO-HIIT, n = 23). The interventions lasted 14 days. **Results:** While circulating markers analysis revealed more protective potential against resorption in HIIT than in WBC alone or combined, gene expression from *in vitro* analysis showed an induction of late bone metabolic markers in the HIIT group. **Conclusions:** These data suggest a potentially protective effect of HIIT in bone against resorption, while WBC maintains homeostasis by preventing any resorptive phenomena and limiting any anabolic activity even when stimulated by intensive exercise.

## 1. Introduction

The therapeutic effectiveness of cold in relieving pain and reducing inflammation is largely known [1], and different applications, such as winter swimming or snow baths, have become popular. In the last 50 years, the use of whole-body cryotherapy/cryostimulation (WBC) has been introduced into clinical practice [2]. WBC is performed in special, controlled chambers (cryochambers) and consists of whole-body exposure to dry air at cryogenic temperatures (≤−110 °C). It has shown positive effects on metabolic profile, low-to-moderate chronic inflammation, and related disorders (e.g., obesity) [3,4], as well as on the treatment of rheumatologic and inflammatory diseases [5]. Cryostimulation has been associated with supporting recovery from injuries or inflammation following extreme effort in athletes, and has become a common practice in sports medicine [6].

It is important to distinguish between “cryotherapy” and “cryostimulation”: the former, an actual therapy to treat painful symptoms, the latter, an adjuvant therapy to improve metabolic/inflammatory responses and to enhance recovery from exercise and injuries [6]. In light of these differences, we refer to WBC as whole-body cryostimulation.

WBC has proven effective in improving the systemic metabolic profile and the homeostasis of tissues involved in energy expenditure and storage, like adipose tissue, skeletal muscle, and bone [7,8]. In the case of bone, in a cohort of rugby players, a combination of WBC with training led to an increase in osteoprotegerin (OPG) when compared to training alone, indicating an osteogenic effect of WBC [8]. In another study, the acute response of a single session of cryostimulation induced an increase in serum sclerostin in physically active young men, indicating an overall negative effect of acute cold stimulation on bone [9]. From this evidence, the molecular effects of WBC on bone metabolism and circulating bone markers need to be further clarified.

In obesity, the dysfunctional adipose tissue shifts toward a pro-inflammatory phenotype, exacerbating overall systemic inflammation. Considering the important anti-inflammatory effects of cryogenic stimulus, WBC has been proposed as adjuvant treatment for obesity [10]. It shows exercise-mimicking and exercise-enhancing effects, and its effectiveness is directly related to the individual percentage of fat mass and to the basal conditions of cardiorespiratory fitness, systemic inflammation, redox profile, and body mass. Additionally, it has been demonstrated that WBC improves metabolic indices (total cholesterol, triglycerides) and metabolic inflammatory mediators related to fat gain (leptin, via β3-adrenergic receptors, resistin, visfatin) [11], causes muscle shivering that induces adipocyte browning through the release of irisin [12], and enhances glucose uptake in muscle and fat [13]. Indeed, it is reasonable that obese subjects, whose condition is characterized by metabolic alterations and chronic low-grade inflammation, could benefit from WBC treatments. However, limited studies have focused on the effects of WBC on obese subjects.

Physical activity (PA) profoundly affects bone metabolism, resulting in bone adaptation to mechanical loads in terms of shape, mass, and strength. Indeed, several studies have associated PA with higher bone mineral density and lower fracture incidence [14]. The beneficial effects of physical exercise are widely recognized, and PA is considered the most effective non-pharmacological therapy for several pathologies that range from obesity to cardiovascular diseases and diabetes [15]. Bone metabolism is differently affected by the kind of PA, according to the intensity, duration, type, and loading degree [15]. PA acts on bone metabolism at different levels: via mechanical load, via the indirect load mediated by muscle traction on the bone, via the endocrine effect mediated by myokines, adipokines, and osteokines, and, as it has been recently defined, via the function of the immune system and modulation of inflammation. Indeed, adipose tissue, skeletal muscle, and bone, thanks to their common embryological origin, are highly interconnected and communicate through a dense crosstalk that affects the others’ functions and together regulate (and are regulated by) innate and adaptive immunity [15]. In particular, the anti-inflammatory and metabolic effects of WBC depend on immune system stimulation, thus mimicking PA [6]. Based on this, WBC, either alone or, possibly more effectively, in combination with PA, could be helpful in the treatment of dysmetabolic conditions, such as obesity and metabolic syndrome and their comorbidities (i.e., osteoporosis and fracture risk).

The aim of this study was to determine the effects of WBC, alone or in combination with high intensity interval training (HIIT), on bone metabolism in obese-to-overweight subjects. This was achieved by analyzing circulating osteo-immune and bone metabolic markers and their potential effects on osteoblast differentiation and function, *in vitro*, by treating SaOS-2 osteoblast-like cells with the sera obtained from the subjects who underwent the different interventions or untreated control. To our knowledge, this is the first study that investigates the effect of WBC and/or HIIT in a population of overweight-to-obese subjects in terms of osteo-immunological markers.

## 2. Materials and Methods

### 2.1. Participants

Inactive, overweight-to-obese participants (n = 67) [body mass index (BMI) = 31.9 ± 5.0 kg·m^−2^, age = 42 ± 13 years] were recruited at Gdansk University of Physical Education and Sport, according to [16]. Inclusion criteria included being overweight or obese, age > 18 years, functionally autonomous, and physically inactive (<60 min/week, and ≤3000 steps/day), as assessed by a questionnaire. Criteria for physical inactivity were obtained with the International Physical Activity Questionnaire (IPAQ), Short Form. Exclusion criteria included taking chronic medications, immune-mediated pathologies, type 2 diabetes mellitus, and traumatic fractures (within 2 years). The study protocol was approved by the Bioethical Committee of the Regional Medical Society (Gdansk, approval #KB-28/17) and conducted in accordance with the Declaration of Helsinki. Participants gave their written consent after being informed about the nature of the study. The sample size was calculated on G*Power (v3.1.9.7), considering as the primary outcome the circulating expression level of Sclerostin and Osteocalcin [9]. Considering the experimental design of four different groups (CTRL group, HIIT group, CRYO group, and CRYO + HIIT group) and two time points (T0 = pre-treatment; T1 = post-treatment), assuming α type I error of 0.05 and a type II error of 0.20 (power = 80%), a minimum sample size of 52 subjects (almost 13 subjects per group) was estimated.

### 2.2. Study Design

Participants performed a maximal exercise test, prior to HIIT, on a recumbent cycle ergometer (ER 900 Jaeger, Germany/Viasys Helth Care and gas analyzer MetaMAx 3B cortex with pre- and post-exercise electrocardiogram recording AsCard Grey), to confirm the absence of any underlying contraindications to participation in vigorous exercise. The test started at 30 W and increased by 15 W/min until volitional exhaustion. Peak power output (W_max_) and maximal heart rate (HR_max_) were recorded. After maximal exercise test clearance by a professional physiotherapist, participants were randomly assigned to one group: control (CTRL, n = 14), training (HIIT, n = 13), WBC (CRYO, n = 17), or training combined with WBC (CRYO-HIIT, n = 23).

Supervised HIIT sessions were performed according to the protocol by Little et al. [17] 3 times/week (Monday, Wednesday, Friday) for 2 consecutive weeks. Training protocol (25 min) consisted of (A) 3 min warm up at 50 W; (B) 10 × 60 s cycling intervals interspersed with 60 s of recovery; and (C) 2 min cooling down at 50 W. The cycle ergometer was set in constant watt mode at 80–100 revolutions/min and individual workloads were set at 90% HR_max_ during the intervals. During recovery, subjects rested by slowly pedaling against a resistance of 50 W. HIIT group performed training in Physiology Department Academy on Physical Education and sport (Gdansk), while CRYO-HIIT group performed exercise training at the Sopot Pomeranian Rheumatologic Centre 1 h before WBC. WBC was performed in an electric cryochamber (ELECPOL, Zimmer Medicine, Sopot, Poland), located in a temperature- and humidity-controlled room. The interventions implied 10 treatments over 2 weeks, with a 2-day rest during the weekend.

Training sessions took place at the same time of day (in the morning, between 8:30 a.m. and 9:00 a.m., after a light breakfast). Blood was collected by a skilled nurse, through standard venipuncture, after overnight fasting, on a 10 min resting subject in a sitting position between 8:00 a.m. and 10:00 a.m. T0 was collected in fasting subjects at the beginning of the intervention (day 1, prior to HIIT and/or cryotherapy). T1 was collected in overnight fasting subjects 24 h after the last intervention.

**Figure 1:**
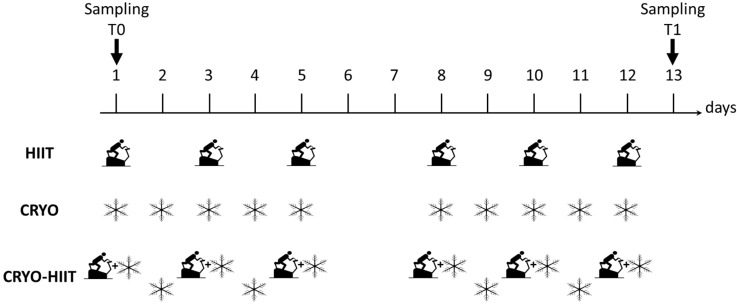
Study design. Scheme of the three intervention groups with indicated the type (HIIT, CRYO or combination of CRYO-HIIT) and frequency of interventions. HIIT was performed 6 times over two weeks (every second day), CRYO was performed 10 times a week over two weeks, while in the combined intervention group, CRYO was performed 5 times a week with combination of CRYO every second day over two weeks. On days 6 and 7 no intervention was performed. Time points of blood collection are also indicated.

During WBC, participants were minimally dressed (bathing suit, socks, clogs, headband, surgical mask); they spent 30 s in a vestibule at −60 °C, to favor adaptation, and then moved to the cryochamber at −110 °C, where they stayed for 3 min. Subjects were under the supervision of skilled personnel. Study design is shown in Figure 1.

### 2.3. Reagents

Unless otherwise specified, products were purchased from Thermo Fisher Scientific (Waltham, MA, USA).

### 2.4. Biomarkers Assessments

Blood samples were collected in fasting subjects as follows. Subjects were allowed to sit for 10 min. A qualified nurse allowed to extend subjects’ arm, and, after vein localization, a tourniquet was applied. After cleaning the collection site with alcohol solution at 70%, standard venipuncture was carried out with a 21 G × 0.75 in BD Vacutainer^®^ Safety-Lok™ Blood Collection Set with 12 in. tubing and a pre-attached holder (Becton Dickinson, Franklin Lakes, NJ, USA). Venous blood was collected in serum SSTII-Advance tubes (Becton Dickinson, Franklin Lakes, NJ, USA). After mixing by inverting 10 times, the serum collected in the tubes was allowed to clot, in a vertical position, for 45 min at approximately 20 °C. The tubes were then centrifuged at 2000× *g* at 4 °C for 10 min to separate the serum. Serum collected was aliquoted and immediately frozen at −80 °C until assayed.

Osteogenic markers Dkk1, FGF23, OPN, OPG, sclerostin and RANKL were analyzed in the serum of each subject with a Luminex^®^ Human Discovery Assay (Biotechne, Minneapolis, MN, USA; sensitivity: 50.9 pg/mL, 10.2 pg/mL, 413 pg/mL, 3.62 pg/mL, 7.0 pg/mL and 4.7 pg/mL respectively) and read on a Luminex^®^ MagPix^®^ (Luminex, Austin, TX, USA). Carboxylated (GlaOC) and undercarboxylated osteocalcin (GluOC) were measured in serum using an immunoassay (Takara Bio, Shiga, Japan; sensitivity: 0.5 ng/mL).

### 2.5. Cell Cultures

Human SaOS-2 osteoblast-like cell line (ECACC, Salisbury, UK) was cultured in Dulbecco’s modified Eagle medium (DMEM) supplemented with 10% HyClone™ fetal bovine serum, 2 mM L-glutamine, 1 × 10^5^ U/L penicillin, 0.1 mg/L streptomycin, 0.25 mg/L amphotericin B, and kept at 37 °C in 5% CO_2_ humidified air. Before each experiment, cells were starved in serum-free medium for 24 h and incubated for 48 h in medium supplemented with 20% filtered serum from subjects underwent to CTRL, CRYO, CRYO-HIIT, or HIIT. Alternatively, after starvation, cells were shifted in osteogenic medium (OM, DMEM supplemented with 150 µM ascorbate 2-phosphate, 10 mM β-glycerophosphate, 10 nM cholecalciferol, 10 nM dexamethasone) with 20% filtered subjects’ sera and grown for 7 or 10 days. OM was replaced every second day.

### 2.6. Cell Viability and Proliferation

Cell viability was indirectly assayed by measuring the mitochondrial reducing potential with the AlamarBlue^®^ assay. Briefly, 9600 cells/well were seeded in a 96-well plate and treated with 20% subjects’ sera for 48 h. Then, AlamarBlue^®^ reagent was added and cells were incubated for 4 h; fluorescence emission was measured at λ = 585 nm (excitation λ = 570 nm) with VICTOR^®^ X3 Multilabel Counter (Perkin Elmer, Inc., Waltham, MA, USA).

Cell proliferation was assayed with Cell proliferation ELISA, BrdU (colorimetric) kit (Roche Diagnostics, Basel, Switzerland). Shortly after incubation with 20% subjects’ sera in the 96 well plate, cells were incubated with BrdU labeling solution for 4 h at 37 °C; BrdU incorporation into DNA of proliferating cells was measured.

### 2.7. Osteogenic Differentiation Assessment

Osteogenic differentiation was assessed in terms of alkaline phosphatase (ALP) activity and quantification of calcified extracellular matrix. In both assays, cells were seeded in 24-well plates at a density of 3 × 10^3^ cells/well, starved and grown in the presence of 20% subjects’ sera with normal medium or OM, for 48 h, 7 days, or 10 days.

For the ALP-specific activity assay, cells were washed in PBS, lysed in 0.1% Triton X-100, cytosol content was collected, and protein content was quantified by BCA Protein Assay. ALP activity was assessed colorimetrically through the measurement of the colored product p-nitro phenol (pNP) formed from p-nitro phenyl phosphate (pNPP) [18]. Calcified matrix deposition was assessed by Alizarin Red-S (ARS) staining [19]. Briefly, cells were fixed with 70% ethanol and stained with 40 mM ARS (Merck, Rahway, NJ, USA). Pictures were taken with Panasonic lg3 camera on a CKX41 Olympus (Olympus, Tokyo, Japan) inverted microscope at 20× magnification. Then, ARS was extracted with 10% cetylpyridinium chloride (CPC) (Merck, Rahway, NJ, USA) in 100 mM phosphate buffer at pH 7.0, and absorbance was measured at λ = 550 nm.

### 2.8. Gene Expression Assay

The QuantiGene™ assay allows for the evaluation of gene expression on a Luminex reader directly from cells without RNA extraction or cDNA synthesis. Briefly, specific probes associated with magnetic beads hybridize with their target RNA, allowing the simultaneous detection of multiple transcripts within a single well. Once the target RNAs are captured by the beads, the signals are amplified, and the fluorescence read [20].

SaOs-2 cells were seeded in 96-well plate at a density of 7500 cells/well, starved for 24 h and then treated with 20% subjects’ sera for 48 h. Cells were lysed and supernatants were probed for mRNA with a QuantiGene™ 10-plex Panel. Results (median fluorescence intensity) depend on the amount of target RNAs. After background subtraction, gene expression was normalized on housekeeping genes (TBP, PPIA) to correct for sample preparation, sample inputs, and inter-well deviation. Fold change was calculated for each target gene. Target and housekeeping genes are listed in Supplementary Table S1.

### 2.9. Statistical Analysis

Circulating cytokine levels, measured in at least 10 subjects per group, are figured as box and whisker plots identifying the median value (intermediate line), the 25th and 75th percentiles (box), and the minimum and maximum values (whiskers). All other data are represented as mean ± SD. Cell treatments were performed with sera from 3 subjects per group and normalized on T0 values. Within-group time-dependent changes (T0 vs. T1) were analyzed by repeated two-way ANOVA and Sidak’s multiple comparison tests. Between-group changes, within a time point, were analyzed by ordinary two-way ANOVA and Tukey’s multiple comparison test. Statistical analysis was performed with Prism v6.01 (GraphPad, Boston, MA, USA).

## 3. Results

### 3.1. Effect of Interventions on Circulating Bone-Related Biomarkers

**Figure 2:**
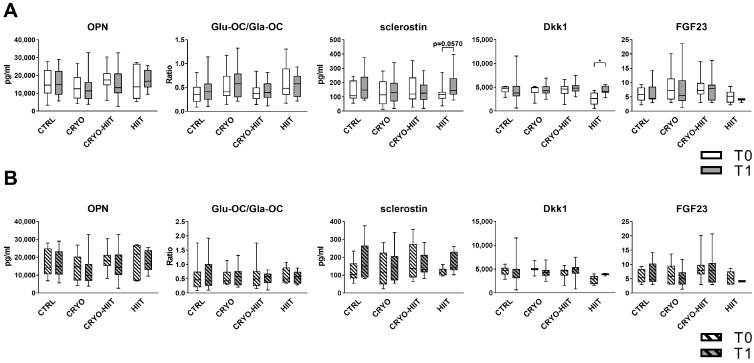
Circulating levels of bone markers in the entire cohort (**A**) and in obese subjects only (**B**). The differences were considered significant when *p* < 0.05. Asterisks indicate significant differences (* *p* < 0.05).

In order to investigate the effects of WBC and/or HIIT treatments on bone metabolism, key circulating bone markers were measured. Neither CRYO, nor HIIT, nor CRYO-HIIT interventions induced any change in the levels of OPN, OC (expressed as GluOC/GlaOC), or FGF23. Sclerostin was slightly induced by HIIT, while CRYO or CRYO-HIIT did not elicit any effect. Dkk1 was modulated only in the HIIT group, being upregulated at T1 vs. T0 (Figure 2A). When stratifying the subjects according to their BMI to select only obese subjects (BMI > 30), no changes were observed in the obese cohort (Figure 2B).

**Figure 3:**
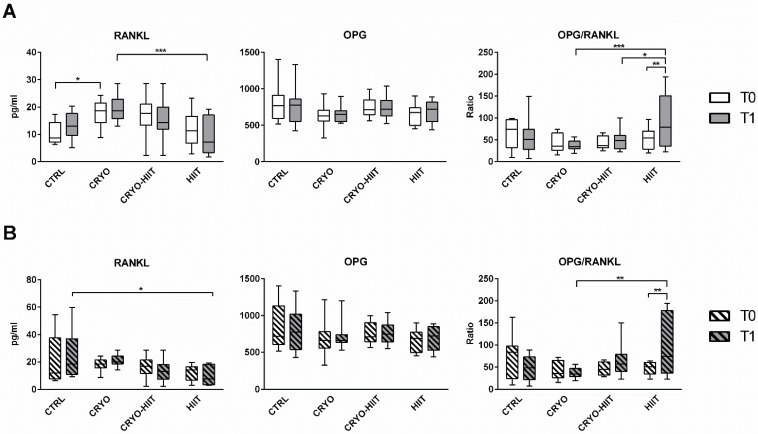
Circulating levels of osteo-immunological markers in the entire cohort (**A**) and in obese subjects only (**B**). The differences were considered significant when *p* < 0.05. Asterisks indicate significant differences (* *p* < 0.05, ** *p* < 0.01, *** *p* < 0.001).

Regarding the osteo-immune compartment, circulating levels of RANKL were significantly upregulated after CRYO treatment at T1 compared to HIIT groups. Curiously, CRYO showed an increase in RANKL circulating levels also at baseline. No within-group differences were observed between T0 and T1. Circulating OPG was unchanged, but the OPG/RANKL was reduced in CRYO and CRYO-HIIT at T1 compared to HIIT and was strongly increased in HIIT group (T0 vs. T1) (Figure 3A). Considering only the obese cohort, RANKL and OPG/RANKL showed no between-group differences at T0, but while RANKL was reduced in HIIT compared to CTRL, OPG/RANKL was increased in HIIT compared to CRYO, at T1 (Figure 3B). Additionally, OPG/RANKL increased in the HIIT group (T1 vs. T0), similarly to the whole population.

### 3.2. Effect of Interventions on Osteoblast Functions in Undifferentiating Conditions

**Figure 4:**
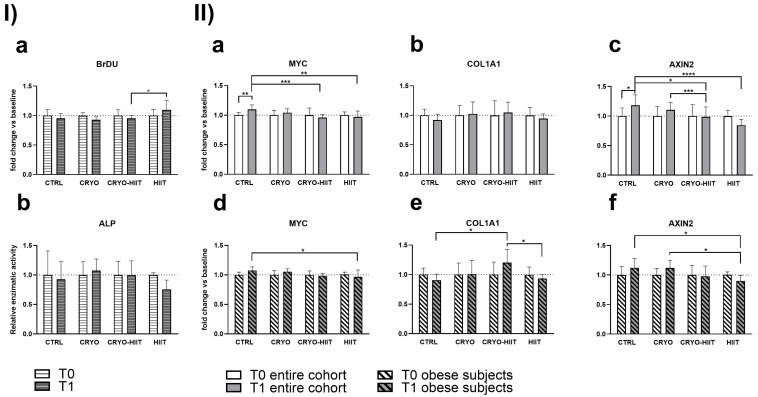
Effect of sera treatment on osteoblast-like cell function. (**I**) Cell proliferation (**a**) and ALP enzymatic activity (**b**) after 48 h and 7 days of treatment with subjects’ sera, respectively. (**II**) Gene expression of significant bone-related genes after 48 h treatment with subjects’ sera, considering the entire cohort (**a**–**c**) or obese subjects only (**d**–**f**). The differences were considered significant when *p* < 0.05. Asterisks indicate significant differences (* *p* < 0.05, ** *p* < 0.01, *** *p* < 0.001, **** *p* < 0.0001).

In order to determine whether 10 WBC sessions, 2 weeks of HIIT, or their combination (CRYO-HIIT) translated to any effect on bone metabolism and osteoblast differentiation potential, osteoblast-like SaOS-2 cells were treated with sera from different groups of subjects for 48 h. Cell proliferation increased significantly in the HIIT group compared to CRYO-HIIT at T1, while no changes were detected between the other groups or as an effect of the intervention (T0 vs. T1) (Figure 4(Ia)). On the other hand, none of the interventions had an effect on ALP activity after 7 days’ incubation with the subjects’ sera (Figure 4(Ib)). Expression of genes related to osteoblast functions was assessed by a QuantiGene gene expression assay on SaOS-2 cells treated with 20% sera from different intervention groups for 48 h. MYC and AXIN2 showed a modulation after treatments (T1) between the different groups: both genes were significantly down-modulated in CRYO-HIIT and HIIT compared to CTRL. A significant downregulation of AXIN2 was observed in CRYO-HIIT compared to CRYO (Figure 4(IIa,c)). All the other genes analyzed remain unchanged (Supplemental Figure S1).

When stratifying the subjects according to their BMI to select only obese subjects (BMI > 30), gene expression of MYC decreased significantly only in HIIT compared to CTRL after intervention (Figure 4(IId)) and AXIN2 decreased significantly in HIIT compared to CTRL and CRYO at T1 (Figure 4(IIf)). COL1A1 showed increased expression in CRYO-HIIT compared to either CTRL or HIIT post-intervention, but only in obese subjects (Figure 4(IIe)). All the other genes analyzed remained unchanged (Supplemental Figure S1).

### 3.3. Effect of Interventions on Osteoblast under Differentiating Conditions

**Figure 5:**
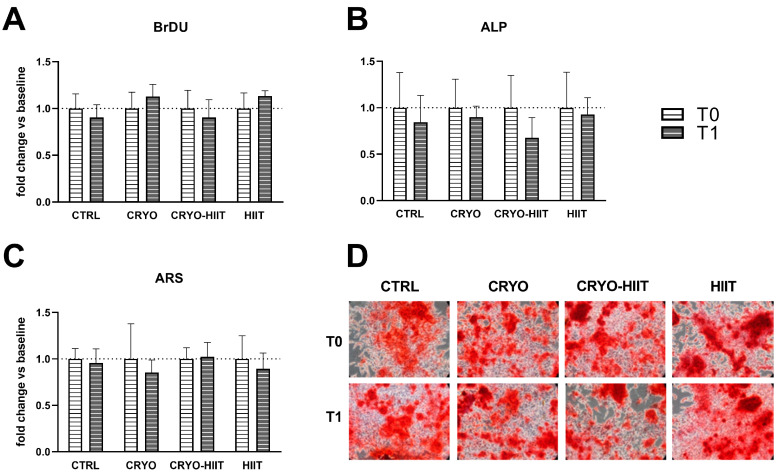
Effect of interventions on osteoblast-like cells under differentiating conditions. Analysis of proliferation (**A**) and alkaline phosphatase (ALP) enzymatic activity (**B**), after 7 days of differentiation, and quantification of calcified extracellular matrix after 10 days of differentiation (**C**,**D**).

The potential effects of HIIT, alone or in combination with WBC, were investigated on differentiated osteoblasts. To this aim, osteoblastic differentiation was induced in SaOS-2 cells during treatments with 20% of subjects’ sera, for 7 or 10 days. Different interventions did not influence cell proliferation and neither had any effect on ALP activity (7 days) or on the deposition of calcified matrix after 10 days (Figure 5).

**Figure 6:**
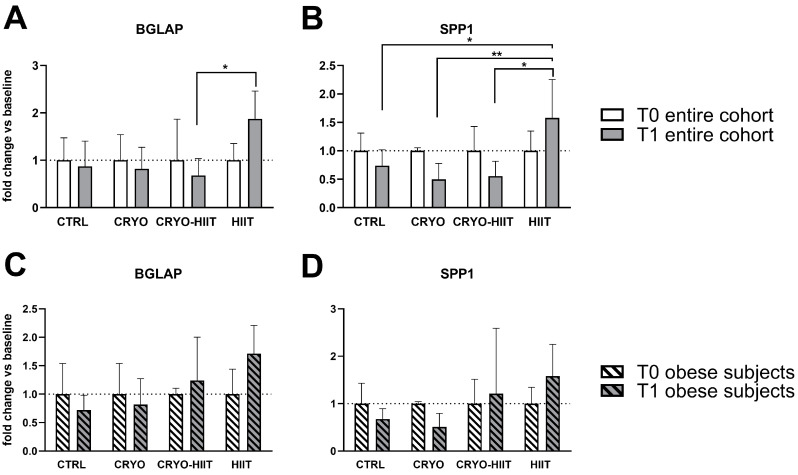
Effect of interventions on bone-related gene expression in osteoblast-like cells treated with subjects’ sera in differentiating conditions. Gene expression of significant bone-related genes after 7 days of differentiation, considering the entire cohort (**A**,**B**) or obese subjects only (**C**,**D**). The differences were considered significant when *p* < 0.05. Asterisks indicate significant differences (* *p* < 0.05, ** *p* < 0.01).

Expression of genes related to osteoblast functions were analyzed also during osteoblastic differentiation. After 7 days of treatments with sera, the expression of RUNX2, MYC, ALPL, COL1A1, and AXIN2 was not modulated in differentiated osteoblasts (Supplemental Figure S2). SPP1 expression was reduced in CRYO and CRYO-HIIT at T1 compared to T0, even though not significantly. However, post-intervention, SPP1 was strongly upregulated in HIIT compared to all the other groups (Figure 6). Similarly, BGLAP was slightly but not significantly reduced in CRYO and CRYO-HIIT, while significantly upregulated in HIIT compared to CRYO-HIIT at T1. All the significances observed were lost when only the obese cohort was considered (Figure 6 and Supplemental Figure S2).

## 4. Discussion

We investigated the effects of WBC, HIIT, and their combination on circulating osteo-immune and metabolic markers of bone and their potential effects on osteoblast differentiation and functions *in vitro*. Although the benefits of PA on bone are nearly undoubtable (according to type, intensity, and duration of PA) [14], the impact of cold-based stimulations, and particularly of WBC, either alone or combined with PA, is still debated.

The hypothesis of a possible effect of WBC on bone is related to the evidences that describe WBC as an exercise-mimicking treatment [6].

Among the circulating markers assessed to investigate the direct effects of the interventions on bone metabolism, only HIIT intervention showed an increase in sclerostin and Dkk1. Although sclerostin and Dkk1 are inhibitors of osteoblast differentiation induced by unloading, they resulted in an increase in those subjects who performed a high-load, high-impact activity like HIIT. This behavior was already reported in professional athletes performing weight-bearing activities (e.g., rugby players) compared to athletes training and competing in unloading conditions (e.g., cyclists) and may be the result of feedback mechanisms aimed at maintaining the coupling between formation and resorption [21]. These results are in agreement with those reported in physically active males subjected to WBC [9] and further supported by the unchanged behavior of FGF23, another unloading-responsive mediator [22]. Moreover, since sclerostin and Dkk1 expression is reported in several cells and tissues, it is possible that changes observed in the HIIT cohort have other functional relevance than the bone response to biomechanical load [23]. The unchanged behaviors of OPN and GluOC/GlaOC indicate that neither the synthetic activity of osteoblasts (OPN, GlaOC, GluOC) nor their engagement in energy metabolism (GluOC/GlaOC) were modified by the interventions.

Cold exposure is also reported to limit the expression of a pro-inflammatory phenotype via the activation of the sympathetic nervous system, as reviewed in Guillot et al. [24]. Inflammation is a key factor involved in bone homeostasis and its importance is, indeed, prototyped by the close link between bone cell differentiation and function and the innate and adaptive branches of the immune system, so much so that it led to the definition of osteo-immune crosstalk. Receptor activator of nuclear factor (NF)-κB ligand (RANKL), a member of the tumor necrosis factor (TNF) superfamily, is a relevant cytokine linking the two systems. RANKL is expressed by a variety of cells (including osteoblasts, other mesenchymal cells, and T-lymphocytes) and, throughout the binding to RANK expressed by pre-osteoclasts and mature osteoclasts, and the consequent activation of NF-κB, it is necessary for the differentiation and function of bone resorptive cells. This system is completed by osteoprotegerin (OPG), another TNF family member, decoy receptor, and inhibitor of RANKL. Additionally, OPG is expressed by osteoblasts and counteracts resorptive potential [25].

In our study, the higher circulating level of RANKL in the CRYO group, compared to HIIT, after completion of the protocol, may result from the higher (although not significant) levels of this cytokine at baseline. Consequently, to the unchanged OPG, OPG/RANKL increased in HIIT, also in the obese sub-cohort. Moreover, OPG/RANKL values in HIIT were significantly higher than those measured for CRYO (also in obese) and CRYO-HIIT after the whole intervention protocol. Overall, these results indicated that the protective potential against resorption is higher for HIIT than for WBC alone or combined with PA. Itpaints a picture in which WBC works by maintaining homeostasis and, in doing so, it does not induce any resorptive phenomenon; at the same time, it limits any anabolic activity even when stimulated by PA.

Adipose tissue acts as an endocrine organ and releases into circulation several factors, called adipokines, that are able to modulate the metabolic response systemically. In addition, in situations of pathological conditions such as obesity, adipose tissue is responsible for a dysregulated secretion of adipokines and other pro-inflammatory cytokines that lead to a systemic state of low-grade inflammation [26]. These circulating factors are able to induce consistent changes *in vitro* as well. It has been demonstrated that osteoblast-like SaOS-2 cells exposed to sera from obese patients are characterized by an altered osteoblastic differentiation [26]. The same research group analyzed the influence of physical activity and hypocaloric diet in obese subjects and found that, in these conditions, SaOS-2 cells treated with subjects’ serum exhibited a significant enhancement in osteoblast differentiation [27]. These findings suggest a positive effect on bone cells of physical activity intervention in obesity. Similarly, in the present study, SaOS-2 cells were treated with serum from subjects of the study groups, either in normal condition or during osteogenic differentiation, to investigate the molecular effects of cold alone or in combination with exercise on osteoblast-like cells. In undifferentiated osteoblast-like cells, treatment with HIIT-serum induced a slight but significant increase in the proliferation rate (vs. CRYO-HIIT, only). Reduction in AXIN2 in HIIT, accompanied by a reduction in c-MYC, a leader of proliferative pathways in osteoblasts [28], and an increase in cell proliferation, indicated that serum from HIIT subjects, either alone or in combination with WBC, has a pro-proliferative effect compared to CRYO treatment alone. Interestingly, the same results were accentuated when considering only obese subjects, who showed also a concomitant decrease in COL1A1 expression. These results are supported by a more substantial, although not significant, decrease in ALP enzymatic activity. These results may be predictors of a reduction in differentiation potential of the HIIT towards a pro-proliferative phenotype in immature osteoblast-like cells. Indeed, Axin2 is a key effector in osteoblast differentiation: it forms complexes with APC (adenomatous polyposis coli) and GSK3β (glycogen synthetase kinase 3β), downstream to the Wnt pathway that controls the activity of β-catenin. When Wnt signaling is induced, GSK3β phosphorylates APC and Axin/Axin2 allowing the release of β-catenin that migrates into the nucleus, where it enters into transcriptional complexes involved in the expression of osteoblastic genes. Other than by phosphorylation, the activity of β-catenin kidnappers depends on their amount and availability into the cytoplasm [29].

On the contrary, during osteoblastic differentiation, pro-proliferating markers remained unchanged, while bone turnover markers, such as BGLAP and SPP1, genes encoding for osteocalcin and osteopontin, respectively, late markers of osteoblastic differentiation, were significantly activated by HIIT intervention, compared to CRYO-HIIT in the entire cohort. A similar trend was observed in ALP enzymatic activity, altogether indicating a counteractive pro-differentiating effect of cryotherapy when combined to HIIT.

Taken together, these results indicate that HIIT, but not cryotherapy or their combination, affects the circulating levels of Dkk1, a bone mechanosensing marker which has a pro-proliferative effect on immature osteoblasts, whereas it enhances osteoblast differentiation in mature osteoblast-like cells.

This study suffers from some limitations. First of all, sample size was quite limited. Second, treatment of SaOS-2 cells with human serum was performed using only three serum samples per group. These samples were chosen considering the best-, mid-, and low-performer for HIIT based interventions (HIIT and CRYO-HIIT) and the best adapting to WBC for CRYO. Thus, these results need to be validated with a larger cohort.

## 5. Conclusions

This study details, for the first time, the effects of WBC alone and combined with a body resistance-based HIIT program in a population of obese-to-overweight subjects. In summary, the results confirm a positive effect of HIIT on bone, with the activation of protective mechanisms against resorption. On the other hand, cryostimulation seems to maintain bone homeostasis: if, from one side, it does not induce any resorptive phenomenon, at the same time, it limits any anabolic activity even when stimulated by intensive PA. In a population of overweight-to-obese subjects, the intervention based on physical activity, and more specifically on HIIT, seems to be more effective than cryotherapy.

## Data Availability

The dataset supporting the conclusions of this article is available in the Zenodo repository: https://zenodo.org/record/8181818#.ZL-RpnZByUk (accessed on 25 July 2023).

## Supplementary Materials

The following supporting information can be downloaded at: https://www.mdpi.com/article/10.3390/jpm14101015/s1, Table S1: List of target and housekeeping genes analyzed in the QuantiGene™ Assay. Figure S1: Gene expression of bone-related genes after 48 h treatment with subjects’ sera; Figure S2: Gene expression of significant bone-related genes after 7 days of differentiation.
