# Supplementary material for: High-Intensity Interval Training, but Not Whole-Body Cryostimulation, Affects Bone-Mechanosensing Markers and Induces the Expression of Differentiation Markers in Osteoblasts Cultured with Sera from Overweight-to-Obese Subjects
Source: J Pers Med. 2024 Sep 24;14(10):1015. doi: 10.3390/jpm14101015 (PMC11508578; doi:10.3390/jpm14101015)
Supplement: Supplementary file 1 [file jpm-14-01015-s001.zip › jpm-3136260-supplementary.pdf]

Supplementary Material

Supplementary Table S1. List of target and housekeeping genes analyzed in the QuantiGene™ Assay.

Gene		Accession number	Encoded protein
RUNX2	Target	NM_001024630	runt-related transcription factor 2 (RUNX2)
COL1A1	Target	NM_000088	collagen type I alpha 1 chain (COL1A1)
SPP1	Target	NM_000582	secreted phosphoprotein 1 (SPP1)
BGLAP	Target	NM_199173	bone gamma-carboxyglutamate protein (BGLAP)
ALPL	Target	NM_000478	alkaline phosphatase, biomineralization associated (ALPL)
MYC	Target	NM_002467	MYC proto-oncogene, bHLH transcription factor (MYC)
AXIN2	Target	NM_004655	axin 2 (AXIN2)
TBP	Housekeeping	NM_003194	TATA-box binding protein (TBP)
RPLP0	Housekeeping	NM_001002	ribosomal protein lateral stalk subunit P0 (RPLP0)
PPIA	Housekeeping	NM_021130	peptidylprolyl isomerase A (PPIA)

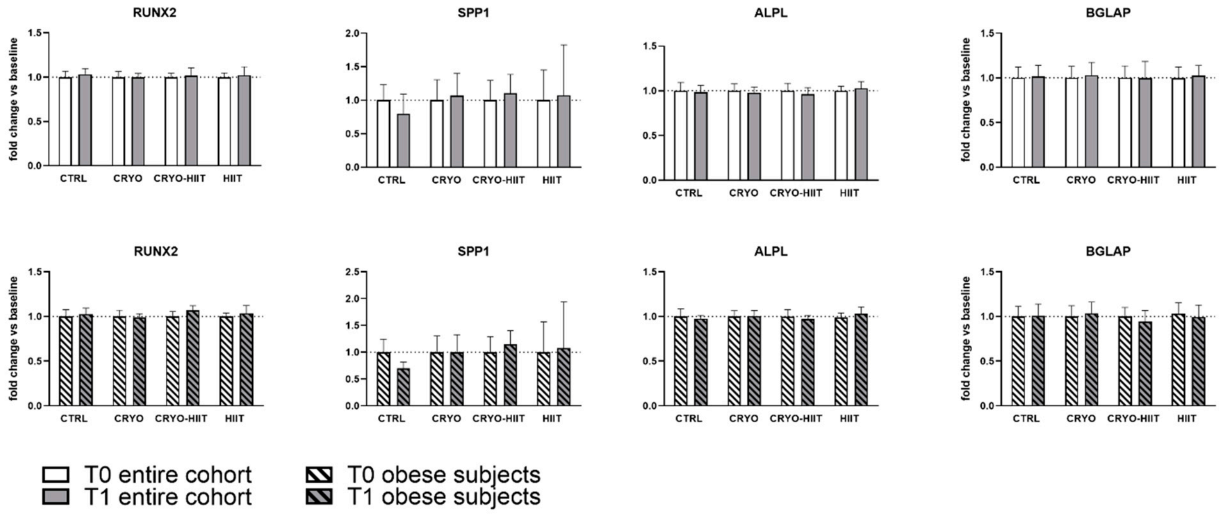

Supplementary Figure S1: Gene expression of bone-related genes after 48h treatment with subjects' sera considering the entire cohort or obese subjects only. The differences were considered significant when $p < 0.05$. Asterisks indicate significant differences (*p< 0.05, ** p< 0.01, *** p< 0.001).

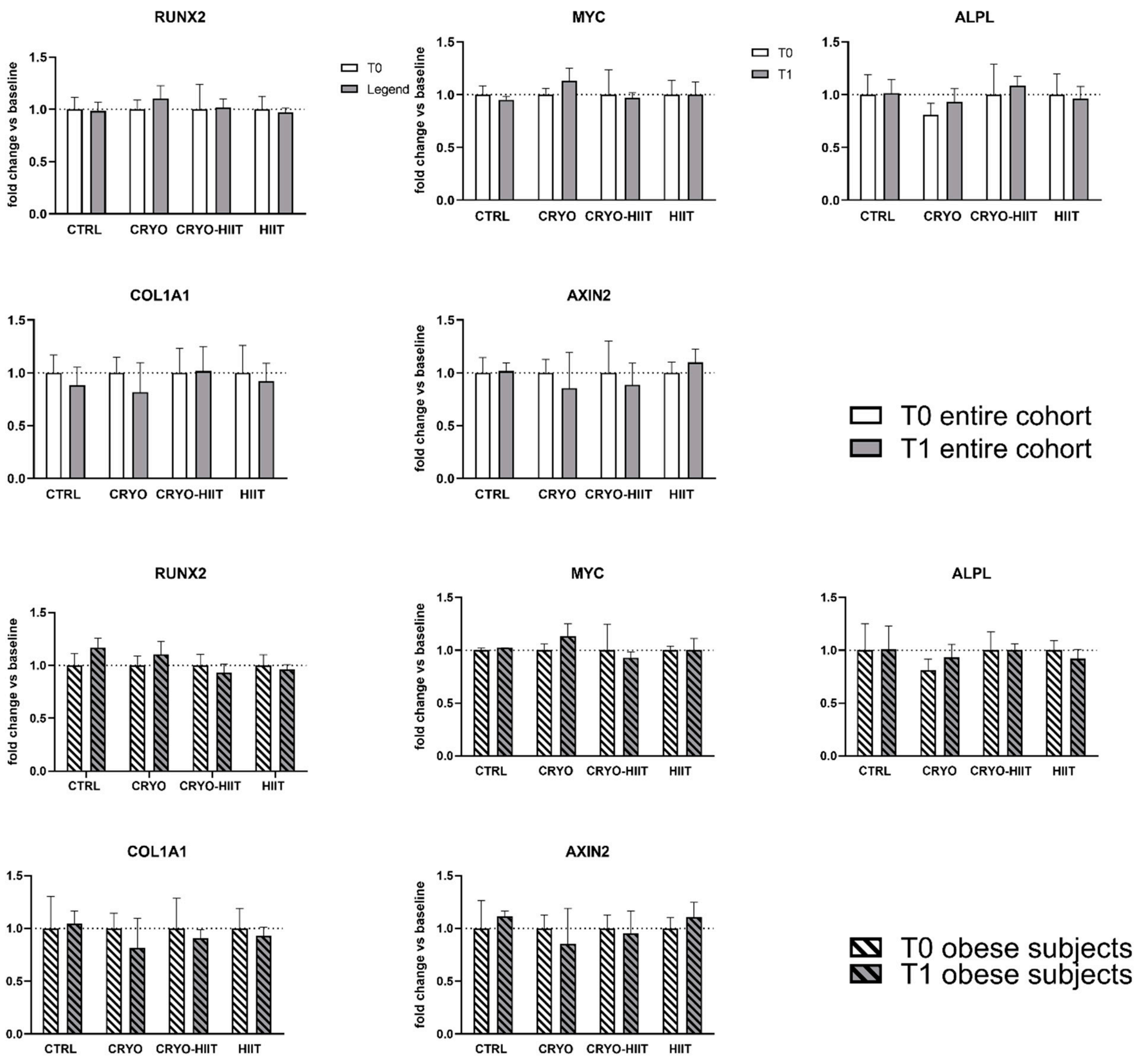

Supplementary Figure S2: Gene expression of significant bone-related genes after 7 days of differentiation, considering the entire cohort or obese subjects only. The differences were considered significant when $p < 0.05$. Asterisks indicate significant differences (*p< 0.05, ** p< 0.01, *** p< 0.001).
